# Cardiac rehabilitation patient perspectives during COVID-19 pandemic: quantitative and qualitative study

**DOI:** 10.3389/fcvm.2024.1373684

**Published:** 2024-07-30

**Authors:** Borut Jug, Natasa Sedlar Kobe, Dejana Stojinic, Mitja Lainscak, Jerneja Farkas

**Affiliations:** ^1^ University Medical Centre Ljubljana, Ljubljana, Slovenia; ^2^ Faculty of Medicine, University of Ljubljana, Ljubljana, Slovenia; ^3^ General Hospital Murska Sobota, Murska Sobota, Slovenia; ^4^ National Institute of Public Health, Ljubljana, Slovenia

**Keywords:** acute myocardial infarction, patients, cardiac rehabilitation, coronavirus disease, pandemic, psychological experience

## Abstract

**Background:**

This study aimed to quantitatively assess stress, anxiety and obsessive thinking related to coronavirus disease-19 (COVID-19) and qualitatively appraise perceptions in patients after acute myocardial infarction (AMI) undergoing cardiac rehabilitation (CR) during the COVID-19 pandemic.

**Methods:**

We used mixed-methods design in patients referred for CR in 2 centres which delivered uninterrupted service during COVID-19 pandemic. Coronavirus Anxiety Scale (CAS), Obsession with COVID-19 Scale (OCS), COVID-19 Stress Scale (CSS), Hospital Anxiety and Depression Scale (HADS), and in-person interviews (combination of *a priori* questions and probing) were used to evaluate patient experience and perceptions with COVID-19 and the healthcare services during pandemic.

**Results:**

In total, 109 patients (mean age 59 ± 10, 20% women) were included in quantitative part and in 30 of them we conducted the in-person interviews. About a quarter of patients met HADS threshold for anxiety and depression while CAS and OCS results demonstrated extremely low possibility of coronavirus related dysfunctional thinking (3%) and anxiety (2%). The CSS indicated the most prevalent concerns were related to COVID-19 vaccines safety (60%) and fear of getting infected (60%). During interviews, patients perceived the CR as well as health care providers as safe, trustworthy and with enough support to avoid or manage COVID-19 related health risks.

**Conclusions:**

Overall, patients reported AMI affected their lives more than the COVID-19 pandemic. The COVID-19 related stress and anxiety were relatively low and mostly related to general views of infectious disease. CR was perceived safe and trustworthy in terms of primary disease and COVID-19.

**Lay summary:**

This mixed-method study included 109 patients with acute myocardial infarction who underwent cardiac rehabilitation during the COVID-19 and focused on their experience and perceptions with COVID-19 and the healthcare services during pandemic.

- Patients reported acute myocardial infarction affected their lives more than the COVID-19 pandemic.
- The COVID-19 related concerns were mostly related to general views of infectious disease (vaccine safety, fear of getting infected) whilst cardiac rehabilitation was perceived safe and trustworthy environment during COVID-19.

## Introduction

Cardiac rehabilitation is a complex multifaceted intervention providing exercise training, cardiovascular risk factor control, secondary prevention and psychosocial support (1–3). The majority of cardiac rehabilitation programs provide centre-based services with patients attending at least 36 group sessions over 12 weeks (4, 5); such organisational framework can accommodate enough interventions to improve cardiovascular outcomes, but also provides patients with intensive follow-up and psychosocial support in the immediate post-diagnosis period (3, 6). In Slovenia, a network of cardiac rehabilitation centres (affiliated with eight regional hospitals) has been established in 2017 and provides unifying content and structure of cardiac rehabilitation programs through a uniform national clinical pathway (7, 8).

The coronavirus disease-19 (COVID-19) pandemic, however, severely disrupted the provision of healthcare, including cardiac rehabilitation. Access to cardiac rehabilitation was hindered by diversion of resources, public health guidance and governmental restrictions (9). Responses of cardiac rehabilitation centres in Slovenia varied from complete shut-downs to carefully adapting programs to the challenges of the COVID-19 pandemic, as reported for other countries (10, 11). While promising alternatives, such as home-based and telerehabilitation, are viable options for cardiac rehabilitation (11, 12), their immediate implementation during the pandemic proved challenging (13); most programs, therefore, continued to provide centre-based rehabilitation on-site, with additional provisions for disease control, including ventilation, face coverings, regular testing, and provision of timely COVID-19 related information (4, 5, 14).

The impact of COVID-19 on cardiovascular healthcare was immediately tangible, while its long-term ramifications have yet to emerge in full (15). In the general population, increased sedentary behaviours, unhealthy eating patterns, along with stress and anxiety during the pandemic have been extensively reported (16–18). In patients undergoing outpatient cardiac rehabilitation, the closure of centres resulted in lost opportunities for functional improvements, motivation, supervision and group-based social support (19). Conversely, experiences and psychological well-being of patients participating in cardiac rehabilitation during the pandemic have been less thoroughly addressed (20, 21).

The information about COVID-19 pandemic effects on patient psychological well-being, personal experience, and trust in the health-care system during cardiac rehabilitation is insufficient. We concieved the mixed-methods study to quantitatively assess stress, anxiety and obsessive thinking related to COVID-19 and qualitatively appraise perceptions in patients attending centre-based cardiac rehabilitation during the COVID-19 pandemic.

## Methods

### Study design

The study was conducted from February 2021 to April 2021, with pandemic in Slovenia in its third wave, with restrictive measures at its peak and with vaccination in its infancy (available as of December 2020, primarily reserved for persons at higher risk of infection). We used mixed-methods approach — i.e., a quantitative prospective design with dedicated instruments (for COVID-19-related anxiety, obsessive thinking, stress, generalized anxiety and depression), and qualitative descriptive design (to examine participants' perceptions of their experiences with cardiac rehabilitation during the COVID-19 pandemic).

### Participant selection

Participants were recruited from two cardiac rehabilitation centres in Slovenia (one university and one regional hospital), which provided uninterrupted cardiac rehabilitation throughout the pandemic. Referral to cardiac rehabilitation after acute myocardial infarction followed international and national recommendations and referral pathways. Eligible patients were adults (over 18 years), who had attended at least 5 cardiac rehabilitation sessions between January 2021 and April 2021.

Cardiac rehabilitation was provided through a structured and comprehensive program with 36 sessions adhering to pertinent guidelines. During the pandemic, the programs were adapted to contain the risk of COVID-19 infection in line with national public health guidance, governmental regulations and professional recommendations. Adapted provision included: limiting number of participants per session (maximal 3, at least 30 m^3^ per patient), intensified hygienic provision, mandatory ventilation and/or opening of windows, mask mandates as per government regulations, regular testing when available (at no cost for patients), regular informing of patients on COVID-19 related issues (including promotion of public health recommendations).

### Quantitative methods — data collection and instruments

We collected basic demographic, socioeconomic, risk factors, comorbidities, and pharmacotherapy information. Additionally, patients completed three COVID-19 related questionnaires and the Hospital Anxiety and Depression Scale (HADS).

The Coronavirus Anxiety Scale (CAS) is a reliable instrument (αs >.90) with solid factorial and construct validity, which captures the frequency of dysfunctional anxiety associated with the COVID-19 pandemic through 5 items scored on a 5-point Likert scale from 0 (“Not at all”) to 4 (“Almost every day over the last two weeks”); the total score ranges from 0 to 20 (with the cut-off ≥9 providing 90% sensitivity and 85% specificity). Internal consistency for the scale in the present study was *α* = .83 (22).

The Obsession with COVID-19 Scale (OCS) is a reliable instrument (αs >.83) with solid factorial and construct validity, which captures the frequency of obsessive thinking about COVID-19. Four items are scored on a 5-point Likert scale from 0 (“Not at all”) to 4 (“Almost every day over the last two weeks”); the total score ranges from 0 to 16 (with the cut-off ≥7 providing 81%–93% sensitivity and 73%–76% specificity). Internal consistency for the scale in the present study was *α* = .88 (23).

A dedicated COVID-19 Stress Scale (CSS) was constructed examining the relevant literature and existing COVID-19-related scales (24); with 19 items, various kinds of concerns about the virus over the past month are investigated and scored on a 5-point scale from 0 (“Not at all”/“Never”) to 4 (“Extremely”/“Almost always”). Following the factor structure of the standard COVID-19 Stress Scale (25), items capturing COVID-19 related fears of becoming infected (4 items), perceived risk of infection when coming into contact with possibly contaminated objects or surfaces (4 items), compulsive checking and reassurance regarding possible pandemic related threats (6 items) pertinent to patients undergoing cardiac rehabilitation were selected. Additionally, 5 items capturing COVID-19 related health-care consequences were constructed. The total score ranges from 0 to 76. The current study yielded acceptable to good reliability coefficients for each of the three subscales (Danger/Contamination fears, *α* = .92; Checking/Reassurance seeking, *α* = .76; Health-care consequences, *α* = .73) and the overall scale (*α* = .91). Confirmatory factor analysis in the Mplus 6 program (26) using the WLSMV estimator indicated that the proposed 3-factor model (Danger/Contamination fears, Checking/Reassurance seeking, Health-care consequences) fits better than the 1-factor model. All factor loadings for the model were greater than 0.50 (see Appendix 1).

HADS is a valid and reliable instrument, which captures symptoms relating to generalised anxiety (HADS-A subscale) and anhedonia, central aspect of depression (HADS-D) (28, 29). Fourteen items (7 for each subscale) are scored on a 4-point Likert scale from 0 to 3, with each subscale score ranging from 0 to 21. Values 8–10 and ≥11 indicate possible and probable mood disorder, respectively; the minimal clinically important difference for cardiac patients is estimated at 1.7 points (30).

### Qualitative methods — interviews

We aimed to interview 15%–20% of included patients, with sample size determined by data saturation criteria (ie. no new categories obtained in the last two interviews). Two investigators (D.S., N.S.) conducted 30–60 min in-person interviews with patients attending cardiac rehabilitation. A semi-structured interview used *a priori* questions (Table 1) and probing (31) to identify perceptions of cardiac rehabilitation during pandemic.

**Table 1:** Interview sample questions.

What you think and how you feel about your experience of acute myocardial infarction? How was it impacted by the pandemic? How was your experience in cardiac rehabilitation program impacted by the pandemic? How was your disease self-management impacted by the pandemic? How were your regular activities and social supports impacted by the pandemic? What were your experiences with the healthcare system during the pandemic? What coping mechanisms you utilized to mitigate this?

### Data analysis — quantitative arm

Data were appraised for normality of distribution visually and formally (Shapiro Wilk test). Summary descriptive statistics are expressed as means [with standard deviation (SD)] or medians [with interquartile range (IQR)] for normally and non-normally distributed continuous variables, respectively, and as total numbers (with proportions) for categorical variables. Comparisons were assessed by *t*-test, Mann–Whitney *U*-test or Chi-square tests, as appropriate.

Possible predictors of COVID-19 scales scores (i.e., CAS, OCS and CSS) were analysed using ordered logistic regression models given the ordered non-interval responses scorings on individual items and assuming proportional odds (formally tested with the Brant test). Ordered regression null mixed-model was fit for interclass coefficient appraisal of possible significant difference between centres, suggesting <2% variation in COVID-19 scales scores was attributable to recruitment centre. COVID-19 multivariate ordered logistic regression model was then fitted with data for the overall population to assess the impact of the total number of comorbidities and social characteristics, and HADS scores on the total CAS, OCS and CSS scores. Statistical significance was set at two-tailed *p *< 0.05. Statistical analyses were performed with Stata/IC 14.2 for Mac (StataCorp, College Station, TX, USA).

### Data analysis — qualitative arm

Interviews were audiotaped and transcribed verbatim. The transcripts were analysed using content analysis (32, 33) as previously described (31), consisting of line-by-line coding and grouping codes into larger categories. Transcripts were independently reviewed by 3 investigators (D.S., N.S., J.F.). After an initial reading of all available transcripts, relevant parts were extracted and preliminarily coded (i.e., given descriptive labels). According to similarities and differences the codes were grouped into the higher order meaning units (subcategories) and named using words that characterize their content. These were organized into core categories. Identification of new contents and inconsistencies in the coding scheme were discussed by a research team and the coding scheme was adapted accordingly. Double-coding of some interviews (*n* = 7) was used to test interrater agreement.

## Results

### Quantitative arm

In total, 109 patients after AMI, all after percutaneous coronary intervention with stenting, were included in a quantitative cross-sectional study during their attendance of cardiac rehabilitation program (49 in General Hospital Murska Sobota and 60 in University Medical Centre Ljubljana, mean age 59 ± 10 years, 20% women) – Table 2. There were no significant differences between the two centers, except in some socioeconomic determinants of health and prevalence of reported comorbidity. All patients were vaccinated during the cardiac rehabilitation unless there were medical or personal constraints against it.

**Table 2:** Characteristics of study participants.

		Overall (*N* = 109)	GH Murska Sobota (*N* = 49)	UMC Ljubljana (*N* = 60)	*p*-value
Demographic	Age, median [IQR]	60 [52–65]	61 [57–67]	60 [52–65]	0.084
	Gender, male %	87 (80)	39 (80)	48 (80)	0.087
Social	Living with partner, *n* (%)	80 (73)	40 (82)	40 (67)	0.079
	School (less than high), *n* (%)	50 (46)	30 (61)	20 (33)	**0** **.** **004**
	Employed, *n* (%)	49 (45)	19 (39)	30 (50)	0.241
	Working class, *n* (%)	42 (39)	28 (57)	14 (23)	**0**.**003**
Medical	Hypertension, *n* (%)	75 (69)	31 (63)	44 (73)	**0**.**019**
	Dyslipidaemia, *n* (%)	85 (78)	30 (61)	55 (92)	**<0**.**001**
	Diabetes, *n* (%)	24 (22)	11 (23)	13 (22)	0.921
	COPD/Asthma, *n* (%)	9 (8)	4 (8)	5 (8)	0.968
	Hx of mental illness, *n* (%)	12 (11)	5 (10)	7 (12)	0.971
Medication	Antithrombotic, *n* (%)	109 (100)	49 (100)	60 (100)	-
	Lipid-lowering, *n* (%)	105 (96)	48 (98)	57 (95)	0.414
	Beta blocking, *n* (%)	105 (96)	47 (96)	58 (97)	0.836
	ACE/AR blocking, *n* (%)	95 (86)	44 (88)	51 (85)	0.100
Questionnaires	CAS sum, median [IQR]	0 [0–1]	0 [0–1]	0 [0–1]	0.402
	CAS ≥9 cut-off, *n* (%)	2 (1.8)	0 (0.0)	2 (3.3)	0.199
	OCS sum, median [IQR]	0 [0–2]	0 [0–2]	0 [0–2]	0.626
	OCS ≥7 cut-off, *n* (%)	3 (2.8)	0 (0.0)	3 (5.0)	0.114
	CSS score, median [IQR]	11 [4–19]	12 [2–20]	11 [5–18]	0.481
	CSS score normalised, mean (SD)	17.1 (14.5)	15.6 (13.2)	18.4 (15.5)	0.426
	HADS – anxiety, median [IQR]	3 [2–6]	4 [2–7]	3 [2–6]	0.866
	HADS – anxiety ≥7 cut-off, *n* (%)	26 (23.8)	13 (26.5)	13 (21.7)	0.555
	HADS – depression, median [IQR]	4 [2–6]	4 [1–6]	4 [2–7]	0.063
	HADS – depression ≥7 cut-off, *n* (%)	27 (24.7)	9 (18.4)	18 (30.0)	0.164

ACE/AR, angiotensin-converting-enzyme/angiotensin receptor; CAS, coronavirus anxiety scale; CSS, COVID-19 stress scale; COPD, chronic obstructive pulmonary disease; GH, general hospital; HADS, hospital anxiety and depression scale; AMI, acute myocardial infarction; OCS, obsession with COVID-19 Scale; UMC, University Medical Centre.

Twentyseven (25%) and twentysix (24%) patients reached the value that more likely indicates depression or anxiety on the HADS questionnaire. An OCS total score of ≥7 was observed in 3 patients (3%) to indicate probable dysfunctional thinking about COVID-19; a CAS total score of ≥9 was observed in 2 patients (2%) to indicate probable dysfunctional coronavirus-related anxiety.

**Figure 1:**
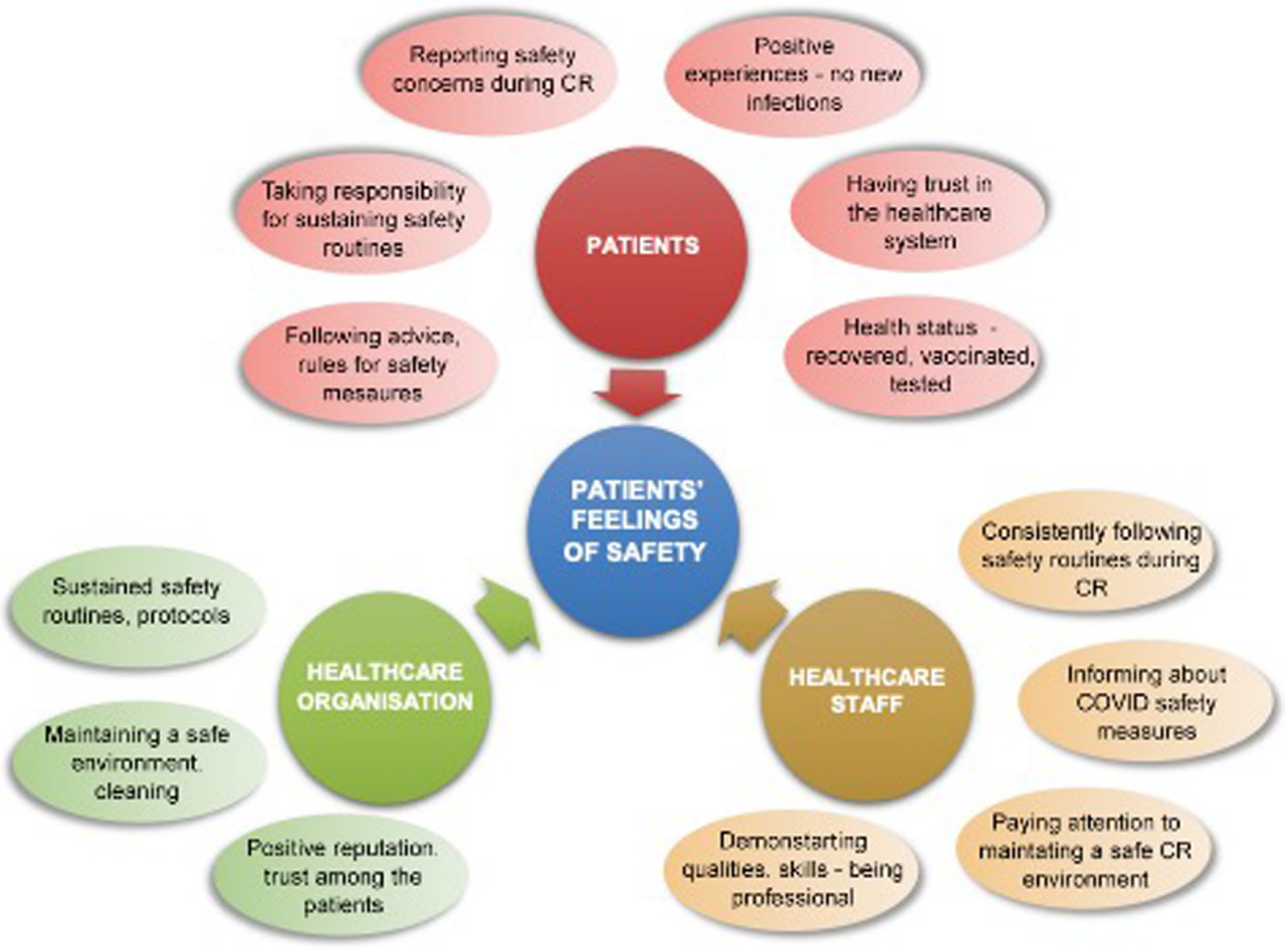
The COVID-19 stress scale (CSS). For items 1–13: How often have you experience following kinds of worries during the last month? 0 (Not at all), 1 (Slightly), 2 (Moderately), 3 (Extremely), 4 (Very often).For items 14–19: During the past month, how much have you done the following because of concerns about COVID-19? 0 (Never), 1 (Rarely), 2 (Sometimes), 3 (Often), 4 (Almost always).

Based on CSS questionnaire results, the level of stress about the COVID-19 among patients attending cardiac rehabilitation program was not high (Figure 1). Patients were most often afraid of getting infected (60%) and had worries about COVID-19 vaccines safety (60%).

**Figure 2:**
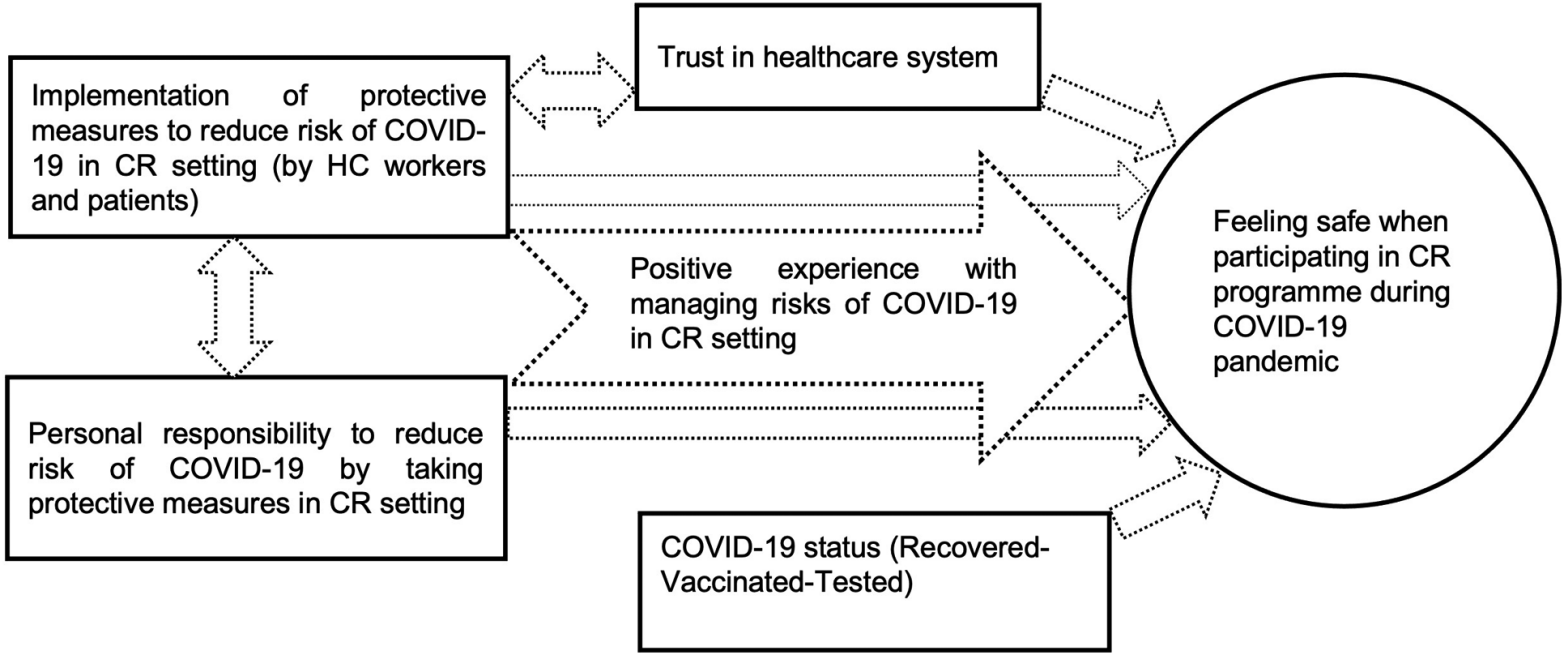
The coronavirus anxiety scale (CAS). How often have you experienced the following activities over the last 2 weeks? 0 (Not at all), 1 (Rare, less than a day or two), 2 (Several days), 3 (More than 7 days), 4 (Nearly every day over the last 2 weeks).

**Figure 3:**
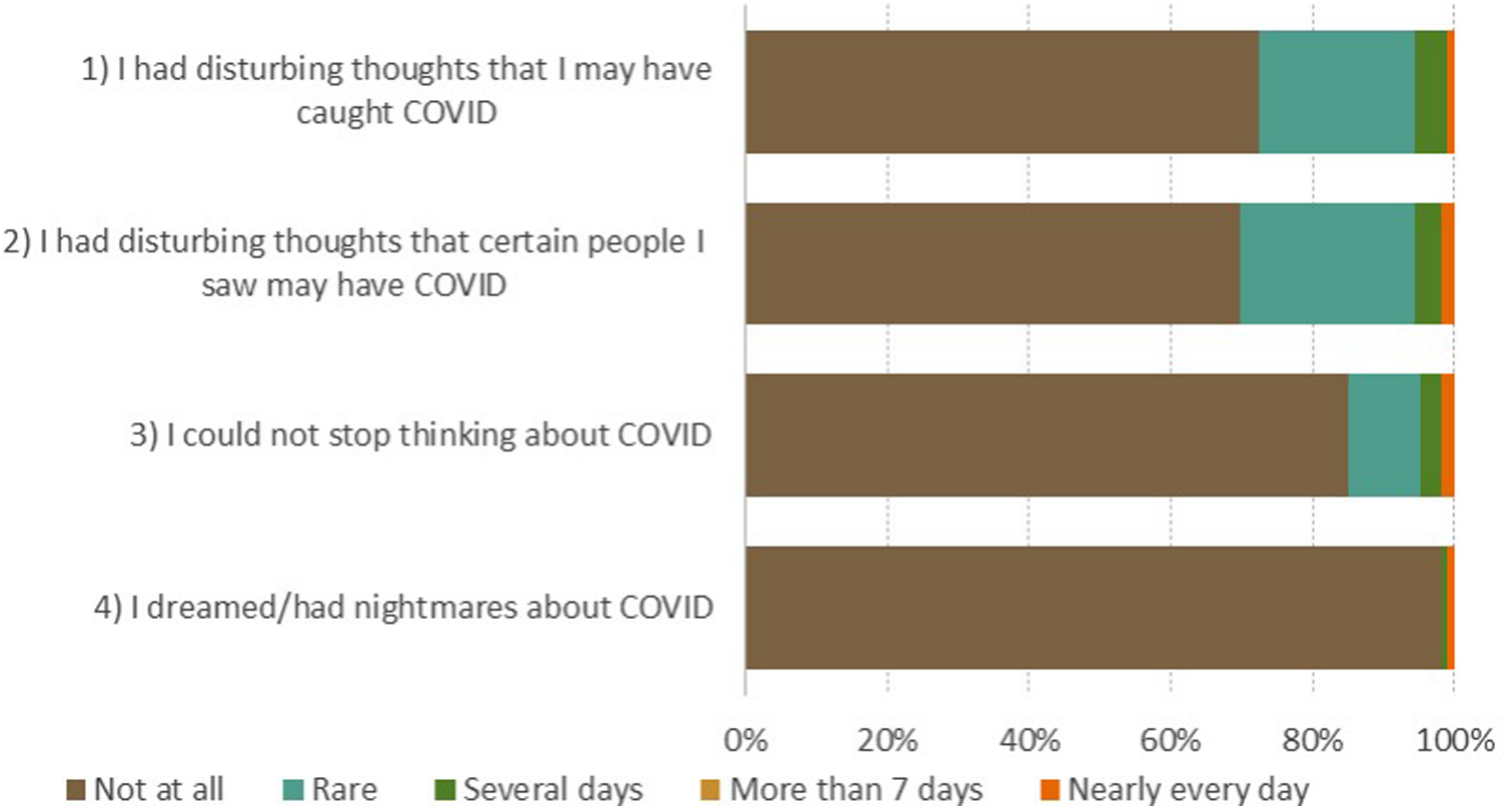
The obsession with COVID-19 scale (OCS). How often have you experienced the following activities over the last 2 weeks? 0 (Not at all), 1 (Rare, less than a day or two), 2 (Several days), 3 (More than 7 days), 4 (Nearly every day over the last 2 weeks).

CAS and OCS scale results (Figures 2, 3) show that patients most often expressed fear of meeting an infected person (30%) and reported sleep problems due to thinking about the coronavirus (15%).

### Qualitative arm

Thirty patients after AMI (15 in General Hospital Murska Sobota and 15 in University Medical Centre Ljubljana) were interviewed in qualitative study of the project. Patients were 59 ± 12 years old, 30% women, 40% employed, majority were living with partner (67%) and 60% had less than high school education (Table 3).

**Table 3:** Psychological experience of COVID-19 pandemic on acute myocardial infarction experience.

Theme	Subtheme	Example quote
Not affected by COVID-19	No additional stress (*n* = 15)	*It was normal … not more difficult due to pandemic … there was no additional stress* (MS, female, 79 years)
	Significant concerns due to AMI, not COVID-19	The experience of AMI is personal (not affected by external circumstances) (*n* = 3) *A heart attack is a heart attack, regardless of an pandemic* (LJ, male, 52 years) Preoccupation with AMI (*n* = 2) *When you experience infarction, you forget about other things happening around (LJ, male, 55 years)* Adaptation to living with AMI not COVID-19 (*n* = 26) *Because of AMI, not because of pandemic, I quit smoking and adjusted my diet (LJ, female, 50 years)*
Stress/tension (*n* = 4)		*/It was/more tense in the beginning … /There was/, strict regime, masks, disinfection … (MS, male, 54 years)*. *I think that if it wasn't for the pandemic, it would be easier to get over the heart attack and all the events surrounding it … because of corona and all this stress it is even more burdensome (LJ, male, 56 years)*
Fear/worry/nervousness	Fear of becoming infected with COVID-19	In the hospital (*n* = 2) *I was afraid of getting sepsis or covid when staying in the hospital* (MS, male, 71 years) In everyday life, especially in crowded spaces (*n* = 9) *I went shopping less often … was afraid I could get infected (MS, male, 54 years)* In social interactions with family, relatives, friends (*n* = 13) *… I didn't meet with my friends because we couldn't know if we were infected (MS, male, 60 years)*
	Fear of not being able to access healthcare provider when needed (*n* = 1)	*I was afraid whether the doctor could come or I could get to him when it/infarction/happened* (MS, female, 60 years)
	Response to threat - trying to reduce risk of COVID-19 by taking protective measures	Taking protective measures when hospitalized (*n* = 6) *I was afraid, I could catch it/covid/in the hospital. I was careful, I wore a mask, I cleaned everything when I went to the toilet (MS, female, 60 years)* Taking protective measures in everyday life [i.e., wearing masks, disinfecting hands (*n* = 11), avoiding crowded places (*n* = 8)] *Mostly I was doing everything as before, but I wore a mask, disinfected hands (MS, male, 80 years)* *My husband and children went shopping for me (LJ, female, 52 years)* Taking protective measures in social interactions with family, relatives, friends[i.e., meeting with less people, isolating, keeping distance, wearing masks when interacting (*n* = 18)] *Only my daughter and grandchildren come to visit, we keep our distance (MS, male, 73 years)* *I wear a mask at home when relatives come to visit (MS, male, 80 years)*
Frustration	Wearing masks (*n* = 6)	*We have to wear masks/in hospital/, that's what bothers me the most (LJ, male, 63 years)*
	Not being able to engage in usual activities outside home due to COVID-19 restrictions (*n* = 12)	*Most difficult was, when I had to be at home/due to movement restrictions/(LJ, male, 58 years)* *I couldn't go to gym as before (LJ, male, 29 years)*
	Difficulties in accessing and communicating with primary health physician	Difficulties in accessing primary health physician (*n* = 12) *There is no personal contact with the doctor. When you call there, no one answers, you get nervous. You call for a couple of days before anyone picks up the phone … if you have a problem, you can't go to the doctor at all (LJ, male, 58 years)* No in-person appointments with primary health physician (*n* = 6) *I miss personal contact, so I can tell him personally what is going on with me (LJ, male, 29 years)*
Social isolation/loneliness	Hospital visitor restrictions (*n* = 4)	*When I was in the hospital, it was difficult for me - you're locked up, you've been through something difficult, you don't have any friends, family around … it was difficult because there were no visits, but luckily, we were able to call each other* via *video call (LJ, male, 56 years).*
	Lack of social support (when needed) (*n* = 10)	*The experience is more difficult due to the lack of social interactions … I felt as if the whole system collapsed, had panic attacks because of loneliness (MS, female, 57 years)*. *I miss going out for coffee with someone, to step back, talk (LJ, male, 54 years)*

AMI, acute myocardial infarction; MS, Murska Sobota; LJ, Ljubljana.

Generally seen, patients attending the interview were similar to others (*p* > 0.1) in all characteristics except for diagnosed hypertension (53% vs. 75%, *p* = 0.032) and dyslipidemia (90% vs. 73%, *p* = 0.063) – see Supplementary Table S1. There were also no differences when comparing CAS, OCS, CSS scores and HADS questionnaire. However, more patients who were not interviewed had HADS score indicative of anxiety and depression.

The interviews yielded two overarching domains: general psychological experience of AMI during the pandemic and psychological experience when visiting cardiac rehabilitation during the pandemic.

### General psychologic experience of acute myocardial infarction during the pandemic

Five major themes, capturing general psychological experience of AMI during the pandemic, emerged from the analysis of interviews: Not affected by COVID-19, Stress/tension, Fear/worry/nervousness, Frustration, Social isolation/loneliness (see Table 3 for themes, subthemes and example quotes).

Patients generally expressed they were not affected by COVID-19. However, emotions that were most experienced during the COVID-19 pandemic were fear, worry, nervousness; participants worried about being infected with COVID-19 and of not being able to access healthcare provider when needed. In response to the threat of COVID-19 infection they tried to reduce its risk by taking protective measures. Some noted that COVID-19 pandemic presented and additional stress during their AMI experience. Frustration due to mandatory masks, limitations due to COVID-19 restrictions and difficulties in assessing primary health physicians was also mentioned. Another negative aspect during patients' AMI experience was feeling of social isolation in the hospital due to hospital visitor restrictions and lack of social support.

### Psychological experience in cardiac rehabilitation program during the pandemic

When asked about their experience in cardiac rehabilitation program during COVID-19 pandemic majority of patients (*n* = 28) reported they had little or no health-related concerns when participating in the program. Some of them noted (*n* = 6) they experienced some fear of COVID-19 infection before or at the beginning of the program.

**Figure 4:**
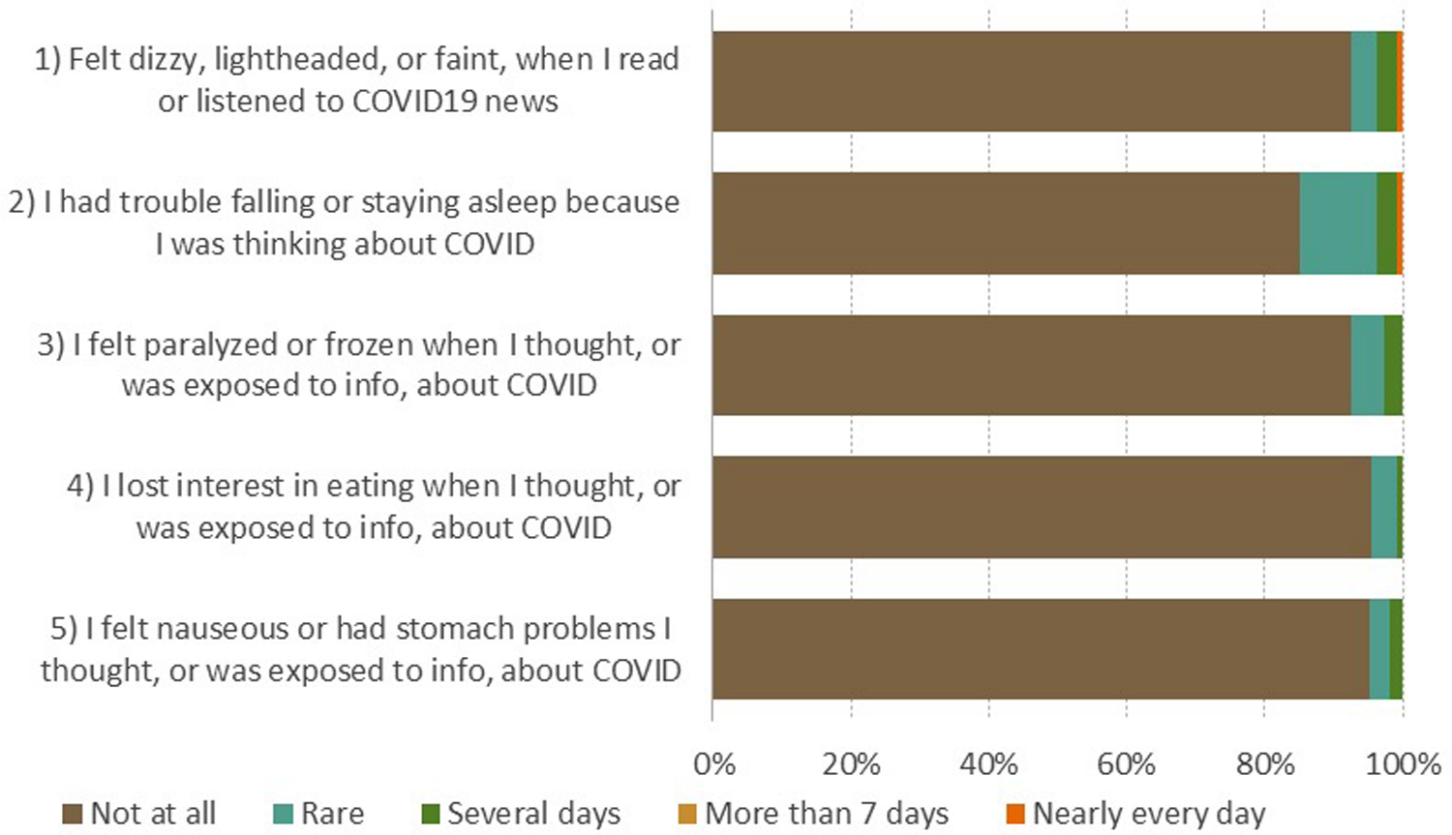
Factors contributing to patients’ feeling of safety when participating in cardiac rehabilitation programme during COVID-19 pandemic identified in the qualitative analysis.

Content analysis of interviews revealed five major themes of factors, contributing to patients' feeling of safety when participating in cardiac rehabilitation program during COVID-19 pandemic: Implementation of protective measures to reduce risk of COVID-19 by healthcare workers and patients in cardiac rehabilitation setting, Personal responsibility to reduce risk of COVID-19 by taking protective measures in cardiac rehabilitation setting, Positive experience with managing risks of COVID-19 in cardiac rehabilitation setting, Trust in healthcare system/workers, COVID-19 status (Recovered-Vaccinated-Tested) (see Table 4, Figure 4).

**Table 4:** Exploring COVID-19 related factors contributing to patients’ feeling of safety when participating in cardiac rehabilitation program during COVID-19 pandemic.

Theme	Example quote
Implementation of protective measures to reduce risk of COVID-19 in cardiac rehabilitation setting (by healthcare workers and patients) (*n* = 23)	*Everything is taken care of: proper ventilation, the windows are always open, you/healthcare workers/keep your distance and always wear masks (LJ, male, 70 years)* *They check us at the entrance, measure our temperature, everything is as it should be, according to regulations (LJ male, 29 years)* *We followed the protective measures – wore masks when cycling, the control/over safety measures implementation/was good, every week unvaccinated/participants/were tested, some were vaccinated … we strictly adhered to the measures - disinfected our hands, wore masks, kept our distance, had windows open, rooms were disinfected (MS, male, 60 years)*
Personal responsibility to reduce risk of COVID-19 by taking protective measures in cardiac rehabilitation setting (*n* = 11)	*I try to take care of myself, pay special attention to the measures … I keep my distance, even outside of rehabilitation (LJ, male, 56 years)*
Positive experience with managing risks of COVID-19 in cardiac rehabilitation setting (*n* = 10)	*In the beginning I had some concerns; there are a lot of us inside, we breathe quickly, we exercise, we couldn't always keep our distance, there are a lot of people in the corridors … nothing ever happened, no one got sick … we always disinfect our hands and all other safety measures are implemented … then I got used to it (LJ, female, 70 years)*
Trust in healthcare system/workers (*n* = 6)	*I trust the healthcare system, I rely on doctors and nurses to guide me (MS, male, 54 years)*
COVID-19 status (Recovered-Vaccinated-Tested) (*n* = 12)	*I recovered/from COVID-19/and was vaccinated, so it was easier for me (MS, female, 74 years)* *If I haven't recovered from covid-19, it would worry me more, but I wouldn't stop attending/cardiac rehabilitation program/ (MS, male, 51 years)*

MS, Murska Sobota; LJ, Ljubljana.

Figure 4 shows the importance of positive experience with managing risks of COVID-19 in cardiac rehabilitation setting that can be established through the implementation of effective infection protective measures (by healthcare workers and participants) and supporting personal responsibility of patients to reduce risk of COVID-19.

**Figure 5:**
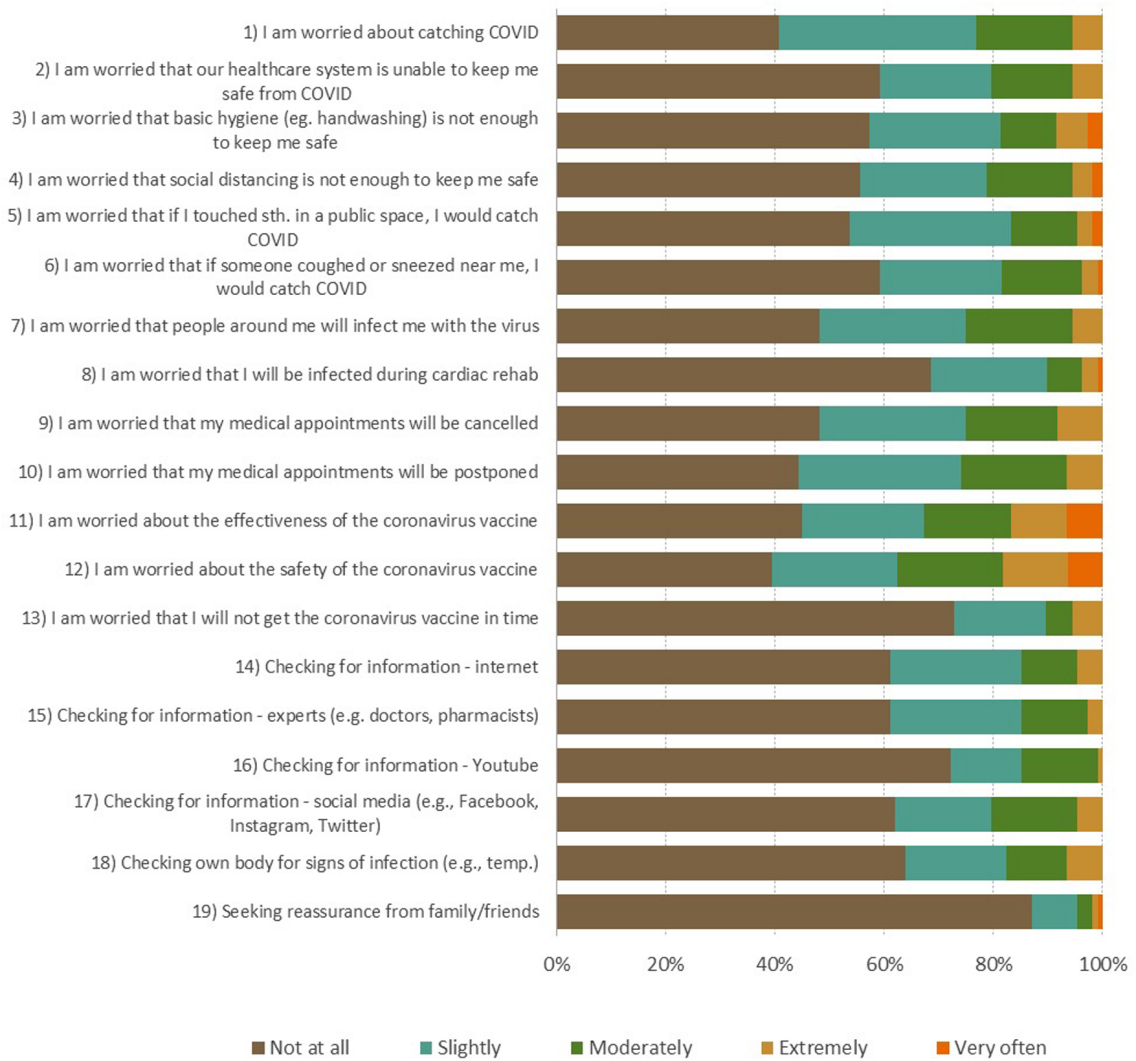
Characteristics of patients, healthcare staff and healthcare organisations that made cardiac rehabilitation patients feel safe during COVID pandemic.

Figure 5 organizes experiences that contributed to patients' feelings of safety when attending cardiac rehabilitation programme during COVID-19 pandemic into characteristics of patients, healthcare staff and healthcare organisations.

## Discussion

In our quantitative and qualitative study about patient psychological well-being, personal experience, and trust in the health-care system in patients after myocardial infarction who attended the outpatient cardiac rehabilitation during COVID-19 pandemic at its peak and lockdown, the reported levels of pandemic related discomfort were relatively low and mostly related to fear of infection and vaccine safety. When analysed in-depth during structured interviews, patients perceived the cardiac rehabilitation program itself as well as health care provider delivery of care as safe, trustworthy and with enough support to avoid or manage COVID-19 health risks.

COVID-19 pandemic and lockdown reduced the delivery of healthcare services in Europe, including access to cardiac rehabilitation (9). In Slovenia, severe restrictions at large as elsewhere were adopted thus only 2 out of 8 cardiac rehabilitation centres were able to meet the required standards and had no interruption in their service. Although the number of patients receiving cardiac rehabilitation was strikingly reduced, the general characteristics were comparable to previous years in Slovenia (8). Both patients and health care providers needed to follow several restrictions in a strict manner that were implemented through medical and governmental authorities. Such restrictions in daily life can result in negative connotation and experience related to healthcare service. As global pandemic started abruptly, there were no specific instruments to address patient needs, experiences and attitudes. The general instruments were initially used while specific instruments were developed at different pace and with different scientific scrutiny. When we conceived this study, only few specific instruments for general population or patients were available therefore we used them along with some established ones to assess these aspects (22–24). As used instruments render quantitative data only, we have additionally performed the interviews in a qualitative study to gain further insights into specific individual characteristics and needs that are beyond the quantitative research. Similar approaches were used previously and also during the COVID-19 pandemics.

To best of our knowledge, the CAS, CSS and OCS were not used in cardiac patients. In fact, only few studies investigated depression and anxiety in cardiac rehabilitation setting during COVID-19 pandemic. According to REACH-HF investigators who focused on heart failure (34), COVID-19 apparently resulted in more anxiety and depression symptoms as assessed by HADS questionnaire. When compared to our results, the average scores in REACH-HF were higher which could also be related to previous observations that patients with heart failure generally have more anxiety and depression than those post myocardial infarction (35). When our findings are compared to previous studies in acute myocardial infarction (36), the psychosocial stress levels were higher than in control subjects but remarkably similar to ours which may reflect that myocardial infarction remained primary patient concern even under COVID-19 pandemic related measures.

With several questionnaires used, we are able to complement the current literature. In our study, patients after AMI expressed low levels of COVID-19 related stress, anxiety and/or obsessive thinking. All measures were associated with general anxiety scores on the HADS-anxiety sub-score domain, but not with demographic, clinical, or socioeconomic characteristics. Regardless, we must highlight those concerns that patients most often reported in connection to COVID-19. On CSS questionnaire, almost two thirds of patients reported worries about COVID-19 vaccines safety and fear of getting infected. Similarly, on OCS questionnaire 30% of patients reported that they had disturbing thoughts that certain people they saw may have the coronavirus. Based on that, it is not surprising that on CAS questionnaire 15% of patients indicated to had trouble falling or staying asleep because they were thinking about the coronavirus. To further appraise perceptions and experiences in patients undergoing centre-based cardiac rehabilitation during the COVID-19 pandemic, we conducted a qualitative study. Results offered insight into psychological experience when visiting cardiac rehabilitation during the COVID-19 pandemic as well as into more general psychological experience of AMI during the COVID-19 pandemic.

As individual experience and perception is relevant when exposed to new situation, the mixed-method with interviews enables us to get in-depth information about patient perception. Others also have used interviews in patients attending cardiac rehabilitation during COVID-19 pandemic yet they focused more on barriers against attendance, which generally were same as prior to pandemic (19, 37, 38). These studies specifically focused on alternatives to institution based cardiac rehabilitation as home based rehabilitation. Patients generally were supportive of this transfer yet additional support to keep motivation at adequate level (e.g., video conferencing, professional supervision) was needed to complete the rehabilitation programme, which can definitely be part of telerehabilitation during pandemics or as a regular service (39).

In our study, we did not have these challenges and patients generally did not report many COVID-19 related issues. A strong theme that however emerged from the data, were changes in psychological experience and lifestyle in relation to experienced myocardial infarction, regardless of COVID-19 pandemic. Similar significant impact of cardiovascular disease on psychosocial outcomes has also been widely noted in literature (40). However, participants in qualitative study noted the impact of their health situation and COVID-19 on fear of becoming infected, resulting in protective behaviour. Similarly, Mejdahl et al. (41) reported that chronic patients who felt they belonged to a particularly vulnerable high-risk group took many precautions with consequences for their everyday life and emotional well-being. We were able to identify factors arising from patients, healthcare staff and healthcare organisations, contributing to patients' feelings of safety when visiting cardiac rehabilitation during COVID-19 pandemic. This is of particular importance as patients needed to visit group rehabilitation sessions amid uncertainty in relation to COVID-19 regularly over a longer period. Sustained safety routines and protocols that are consistently followed by healthcare staff and patients, their mutual efforts to maintain a safe environment, including patients' possibility to report safety concerns, professionalism of healthcare workers, general trust in healthcare system, were identified. Similar safety-related factors arising from a range of care experiences in healthcare setting have been acknowledged by Barrow et al. (42). Our findings could form a basis for future healthcare in uncertain circumstances during pandemics, especially for chronic patients that need regular contact with healthcare system.

Our findings deserve to be interpreted with some caution. Firstly, the sample size may be considered as rather small but when compared to other studies in the field, we are well off in particular in qualitative aspect as our sample was larger than in others. Also, it needs to be pointed out that the study was conducted in all national cardiac rehabilitation centres that were open during the COVID-19 pandemic, which means that we have captured the maximum possible number of patients during pandemic. Secondly, the interviews were conducted between the second and third wave, when the number of patients with COVID-19 was relatively low, and we already had first experience with COVID-19 that might have had affected patient and healthcare professionals' behaviours during next waves. Thirdly, we have included only patients after AMI, which was due to limited cardiac rehabilitation network capacity and need to prioritize patients as per level of guideline recommendation (4, 43). Finally, the clinical diagnosis of anxiety or depression was not confirmed in full.

## Conclusions

Patients reported that the acute myocardial infarction affected their lives more than the COVID-19 pandemic. Patients attending cardiac rehabilitation showed relatively low levels of stress and anxiety due to the COVID-19 pandemic. A higher level of stress, anxiety and obsessive thinking due to the coronavirus was detected in patients with a more pronounced level of anxiety, but not in connection with somatic indicators of cardiovascular disease. It was shown that the consistent implementation of measures to prevent infection in the medical institution, the participants' own concern for the implementation of preventive measures and positive experiences in managing the risks of infection significantly contributed to the feeling of safety when attending the cardiac rehabilitation program during the COVID-19 pandemic.

## Data Availability

The raw data supporting the conclusions of this article will be made available by the authors, without undue reservation.

## Supplementary material

Supplementary Table S1 Characteristics of participants.

## Appendix 1

**Table 1A:** Confirmatory factor analysis factor loading of the proposed CSS model.

Coronavirus Stress Scale (CSS) for patients in cardiac rehabilitation program	Scale	Factor estimate (SE)		
		I	II	III
I am worried about catching COVID-19.	D/C	.81 (.04)		
I am worried our healthcare system is unable to keep me safe from COVID-19.	D/C	.73 (.05)		
I am worried that basic hygiene (e.g., handwashing) is not enough to keep me safe.	D/C	.87 (.03)		
I am worried that social distancing is not enough to keep me safe.	D/C	.88 (.03)		
I am worried that if I touched something in a public space, I would catch COVID-19.	D/C	.90 (.03)		
I am worried that if someone coughed or sneezed near me, I would catch COVID-19.	D/C	.92 (.03)		
I am worried that people around me will infect me with the virus.	D/C	.91 (.02)		
I am worried that I will be infected during cardiac rehabilitation program.	D/C	.80 (.05)		
I am worried that my medical appointments will be cancelled.	HC		.79 (.05)	
I am worried that my medical appointments will be postponed.	HC		.72 (.06)	
I am worried about the effectiveness of the coronavirus vaccine.	HC		.97 (.02)	
I am worried about the safety of the coronavirus vaccine.	HC		.90 (.02)	
I am worried that I will not get the coronavirus vaccine in time (Health-care consequences).	HC		.94 (.06)	
Checking for information – internet (Compulsive checking and reassurance seeking).	CH			.64 (.07)
Checking for information - experts (e.g., doctors, pharmacists).	CH			.74 (.07)
Checking for information – Youtube.	CH			.79 (.07)
Checking for information - social media (e.g., Facebook, Instagram, Twitter).	CH			.52 (.10)
Checking own body for signs of infection (e.g., temperature).	CH			.73 (.10)
Seeking reassurance from family/friends.	CH			.87 (.09)
	Danger/contamination			
Health-care consequences	.670 (.042)			
Compulsive checking and reassurance seeking	.603 (.558)			

Scale: D/C, COVID-19 danger/contamination fears; HC, COVID-19 health-care consequences; CH, COVID-19 compulsive checking and reassurance seeking.

Factor I, Danger/Contamination; Factor II, Health-care consequences; Factor III, Compulsive checking and reassurance seeking.

Confirmatory factor analysis (CFA) was conducted to confirm the scale's factor structure in the Mplus 6 program22 using the WLSMV estimator. The following model fit indices were used besides the chi-square statistic (the approximate cut-off values and value-related references are in parentheses): RMSEA [<.06; (27)]; CFI [>.95, (27)], TLI [>.95; (27)], WRMR [<.90; (26)].

The proposed 3-factor model (danger/contamination fears, checking/reassurance seeking, health-care consequences) had a better model fit [χ^2^(149) = 424.7, *P* < .001; RMSEA = .13, 90% CI: .12–.15; CFI = .93; TLI = .92; WRMR = 1.40] compared with the unidimensional model (i.e., all 19 items loading on a single factor) and [χ^2^(152) = 658.9, *P* < .001; RMSEA = .18, 90% CI: .16–.19; CFI = .86; TLI = .85; WRMR = 1.87], with reliability coefficients alpha of the scales.92,.76 and.73, respectively. All factor loadings for the 3-factor model were greater than 0.50. However, in terms of goodness-of-fit indices the original 5-factor model (25) performed better (RMSEA = .05, 90% CI: .05–.05; SMRM = .04; CFI = .93).
