# Supplementary material for: Cardiac rehabilitation patient perspectives during COVID-19 pandemic: quantitative and qualitative study
Source: Front Cardiovasc Med. 2024 Jul 30;11:1373684. doi: 10.3389/fcvm.2024.1373684 (PMC11319166; doi:10.3389/fcvm.2024.1373684)
Supplement: Supplementary Table S1 — Characteristics of participants. [file Table1.docx]

**Supplementary table 1.** Characteristics of participants

|  |  | Overall  (N=109) | Not taking part in the interview (N=79) | Taking part in the interview (N=30) | P (Taking part vs. not taking part in the interview) |
| --- | --- | --- | --- | --- | --- |
| Demographic | Age, median [IQR] | 60 [52-65] | 60 [54-65] | 59 [52-69] | 0.587 |
|  | Gender, male % | 87 (80) | 66 (84) | 21 (70) | 0.118 |
| Social | Living w/partner n (%) | 80 (73) | 60 (76) | 20 (67) | 0.332 |
|  | School (less than high), n (%) | 50 (46) | 32 (41) | 18 (60) | 0.182 |
|  | Employed, n (%) | 49 (45) | 37 (27) | 12 (40) | 0.988 |
|  | Working class, n (%) | 42 (39) | 28 (35) | 14 (47) | 0.252 |
| Medical |  |  |  |  |  |
|  | Hypertension, n (%) | 75 (69) | 59 (75) | 16 (53) | 0.032 |
|  | Dyslipidaemia, n (%) | 85 (78) | 58 (73) | 27 (90) | 0.063 |
|  | Diabetes, n (%) | 24 (22) | 16 (20) | 8 (27) | 0.475 |
|  | COPD/Asthma, n (%) | 9 (8) | 7 (9) | 2 (7) | 0.310 |
|  | Hx of mental illness, n (%) | 12 (11) | 11 (14) | 1 (3) | 0.196 |
| Medication | Antithrombotic, n (%) | 109 (100) | 79 (100) | 30 (100) | 0.161 |
|  | Lipid-lowering, n (%) | 105 (96) | 75 (95) | 30 (100) | 0.213 |
|  | Beta blocking, n (%) | 105 (96) | 76 (96) | 29 (97) | 0.909 |
|  | ACE/AR blocking, n (%) | 95 (86) | 69 (87) | 26 (87) | 0.705 |
| Questionnaires | CAS sum, median [IQR] | 0 [0-1] | 0 [0-0] | 0 [0-0] | 0.328 |
|  | CAS ≥9 cut-off, n (%) | 2 (1.8) | 2 (0.3) | 0 (0) | 0.540 |
|  | OCS sum, median [IQR] | 0 [0-2] | 0 [0-2] | 0 [0-2] | 0.757 |
|  | OCS ≥7 cut-off, n (%) | 3 (2.8) | 2 (3) | 1 (3) | 0.384 |
|  | CSS score, median [IQR] | 11 [4-19] | 12 [4-22] | 11 [3-15] | 0.876 |
|  | CSS weighted, mean (SD) | 17.1 (14.5) | 18.6 (15.6) | 13.1 (10.0) | 0.889 |
|  | HADS – anxiety, median [IQR] | 3 [2-6] | 3 [2-7] | 4 [1-5] | 0.695 |
|  | HADS – anxiety ≥7 cut-off, n (%) | 26 (23.8) | 23 (30) | 3 (10) | 0.037 |
|  | HADS – depression, median [IQR] | 4 [2-6] | 4 [2-7] | 4 [1-6] | 0.614 |
|  | HADS – depression ≥7 cut-off, n (%) | 27 (24.7) | 24 (30) | 3 (10) | 0.028 |

ACE/AR – angiotensin-converting-enzyme/angiotensin receptor; CAS - Coronavirus Anxiety Scale; CSS - COVID-19 Stress Scale; COPD – chronic obstructive pulmonary disease; GH - General Hospital; HADS - Hospital Anxiety and Depression Scale; AMI – acute myocardial infarction; OCS - Obsession with COVID-19 Scale; UMC - University Medical Centre
